# Islam and Veterinary Science: Rethinking Animal Suffering Through Islamic Animal Ethics and the Evolving Definition of Halal Slaughter

**DOI:** 10.3389/fvets.2022.785585

**Published:** 2022-05-17

**Authors:** En-Chieh Chao

**Affiliations:** Sociology Department, National Sun Yat-sen University, Kaohsiung, Taiwan

**Keywords:** veterinary anthropology, history of veterinary ethics, multicultural history of science, animal ethics and welfare, Islam

## Abstract

In the midst of recent European activism against religious slaughter, the idea that religious slaughter is cruel to animals is often seen as commonsense, and the mandatory pre-slaughter stunning is often portrayed as the moral technology that assures animal welfare. Nevertheless, this portrayal seems to blur the fact that the current notion of animal welfare itself is built upon a changing selection of value assumptions, which are not without problems or academic debates. It also ignores the fact that contemporary veterinary scientists and Muslim scholars have been working together for four decades to learn more about farm animals and their suffering. Despite stereotypes, the idea of animal ethics is not foreign to Islam. In Islam, animals represent God's wisdom and wonder, and humans are obliged to attend to their health and living conditions. When killing animals for food is conducted, the slaughter must be done in the name of God as a sacred ritual in order to assure that the life of the animal is not taken lightly and that the slaughter is not a sign of hostility toward the universe. Before the act of sacrifice, the animal must be healthy, and no harm should be forced upon it. Accordingly, the requirement of pre-slaughter stunning has posed a question to Muslim scholars: Does stunning kill the animal or cause harm? What defines harm, and whose definition counts? This paper reconstructs a socio-technological history of halal slaughter through scientific research on animal suffering since the 1980s. On the basis of archival research of New Zealand veterinary scientists' works and in-depth interviews with Malaysian veterinary scientists, this article outlines three phases of the evolution of halal slaughter that aims to fulfill multiple sets of moral obligations toward farm animals, and demonstrates how veterinary scientists establish common ground between secular and Islamic animal ethics. In this vein, I am envisioning a possibility of veterinary anthropology that recognizes the field's trans-cultural characteristics, and continues to challenge the rigid binaries between the West and the Rest, and between science and culture.

## Introduction

In the midst of recent European activism against religious slaughter, the idea that religious slaughter is cruel to animals is often seen as commonsense, and the mandatory pre-slaughter stunning that renders animals insensible to killing is often portrayed as the moral technology that assures animal welfare. Nevertheless, this portrayal seems to blur the fact that the current notion of animal welfare itself is built upon a changing selection of value assumptions, which are not without problems or academic debates—for example, what counts as suffering? What has intrinsic value worthy of protection (1, 2)? Equally important, the aforementioned dichotomized portrayal ignores the fact that contemporary veterinary scientists and Muslim scholars have been working together for four decades to learn more about farm animals and their suffering. This kind of collaboration represents an excellent example of how it is possible and rewarding to rethink animal ethics from different cultural perspectives.

Here, I am envisioning a possibility of veterinary anthropology that explores the trans-cultural history of scientific veterinary practices. Complementing the emerging field of ethnographic accounts of human-animal health and the political economy of zoonoses (3–6), my approach focuses on the intersection of science, human-animal relations, and the history of veterinary practices in (trans)culturally-specific ways. In this paper, I demonstrate one way this approach can contribute to social studies of veterinary science by narrating a socio-technical history in which the scientific work of veterinary scientists enables the changing definitions of halal slaughter, and Islamic animal ethics also facilitates scientific studies of animal suffering.

The idea of animal ethics is not foreign to Islam. In the heterogenous traditions of Islam, animals represent God's wonder, and humans are obliged to attend to their health and living conditions (7–9). Copious documents regarding the history of veterinary science in Islam show that both ordinary people and veterinarians were instructed to treat animals with kindness (8). For example, Mamluk veterinarians' ethical guidelines contain innumerable descriptions of proper attitudes to animals, all mentioning the moral obligation of humans who take care of animals to treat them compassionately. While we are far removed from medieval times, two rationales justify why we should keep the trans-cultural history of veterinary practices in mind.

First, this history reminds us of the very existence of multiple animal ethics. Moving between different ways of thinking about animal suffering encourages us to not take for granted the standard procedures taken in the modern slaughter house. For example, in an Eid al-Adha festival, Muslims are taught to compassionately deal with the pain of animals not by removing it, but by acknowledging and witnessing it. The Sufi teacher Muhammad Raheem Bawa Muhaiyaddeen (10) describes the duty of the Muslim slaughterer, writing that to “look into the animal's eyes, he has to watch the tears of the animal, and he has to watch the animal's eyes until it dies—hopefully his heart will change.” In contrast, the current system of industrialized animal factories ensures that both the scene of killing and the animal's struggling before death are removed from humans' sight. A complex system of avoidance is set up, in which abattoirs are kept away from everyday life for most people. Even inside the abattoir, the administration of death is broken down into several steps that obscure the question of “who kills the animal,” and “completely dilute the responsibilities and any feelings of guilt” (11). While the pre-slaughter stunning is intended to eliminate animals' feelings at the moment of death, it is also precisely conducted to enhance their “killability,” as their fear and pain would disrupt the smooth operation of industrial slaughter (12). In short, animals are made more “killable” once they lose their sentience and their abilities to feel (13).

Second, an appreciation of different animal ethics not only assists us in reflecting upon the mainstream practice of meat production, but also enables us to explore the mutual influence of cultural and scientific practices. The practices of (sub)culture and science are commonly assumed to be unrelated in the scientific field, but their interdependence is richly documented in the literature of social studies of science (14–17). It is my argument that investigating the trans-cultural history of veterinary science better equips anthropologists to reconceptualize veterinary science as scientific *and* cultural practices that are made and remade in shifting historical contexts.

Bearing in mind the multiple animal ethics and the co-construction of science and culture within veterinary practices, this paper reconstructs an overlooked techno-moral history of veterinary scientists' research of halal slaughter since the 1980s. Halal means “permissible,” and ideally Muslims only consume halal food when circumstances allow. For a slaughtering process to be halal, a number of criteria are to be met. Of those requirements the western imagination is often captivated by the bleeding process, imagining it as a kind of collective, religious hematophobia that drives the slaughter conduct against all considerations of animal ethics. Yet, the truth is that bleeding is also a standard procedure in the mainstream, secular slaughterhouses. More importantly, the imperative of minimizing animal suffering is one of the requirements that constitutes the core of halalness. It is typically achieved by the utilization of an extremely sharp knife aimed perfectly at the spot that can cut the carotid artery, jugular vein, trachea and esophagus all at once in one slice (18). Additionally, the animal must be healthy and without injuries before the slaughter (18, 19). As a result, a carcass or a wounded animal body is also considered as haram or “impermissible” to consume for Muslims. Finally, the slaughter must be done in the name of God as a sacred ritual in order to assure that the life of the animal is not taken lightly and that the slaughter is not a sign of hostility toward the universe (19).

Accordingly, the requirement of pre-slaughter stunning has long posed a question to ulama/mufti or Muslim scholars: Does stunning kill the animal? Does it cause harm? What defines harm, then? Whose definition counts? This paper responds to these questions by reconstructing a techno-moral history of the trans-cultural alliances among veterinary scientists and Muslim scholars. Their goal was to establish common ground between secular and Islamic animal ethics where the animal's insensibility to pain and the animal's protection from harm before the act of slaughter can coexist. As I will show, both secular and Islamic systems of animal ethics have been changing and interacting with each other in this convoluted process.

## Materials and Methods

The research data and analysis presented in this paper is the result of archival research and interviews conducted between 2015 and 2021. The archival materials of technical reports that belong to the Meat Industry Research Institute of New Zealand (MIRINZ) were obtained in 2017 thanks to the generous help of the librarians at AgResearch (formerly known as New Zealand Pastoral Agriculture Research Institute Limited) in New Zealand. Interviews have been conducted since 2019 with veterinary scientists at a research University in Malaysia I call University X, whose cutting edge research that integrates Islamic ethics and veterinary science is illuminating for my research. They will remain anonymous at their request.

One obvious question for readers is the reason for choosing the research of these two groups to study. The reason I focus on the MIRINZ's research is due to the fact that they are the first developer of “halal” stunning that has won international approval in the 1980s. The reasons to also focus on University X's research are 2-fold. First, veterinary scientists at University X have served as scientific advisors to the Malaysian government agency in charge of Islamic affairs, and the Malaysian government has been a leading player in setting halal standards that are followed worldwide, along with the neighboring Indonesian Ulema Council. The reasons behind why these two countries are pioneers of halal certification are complex, which are connected to ethnic and religious identity politics in the region that I have addressed in detail elsewhere (20).

Second, I intend to echo philosopher of science Sandra Harding's call to treat modern sciences as inevitably embedded in cultural values on one hand, and to focus on scientific ideas and practices in non-Western settings, on the other hand (21). This post-colonial approach is one that “does not give up the epistemology of modern science,” but one that sees an exploration of values behind sciences as helpful resources to build a more inclusive, multicultural, and critical history of science and technology. Without falling prey to unhelpful relativism, Harding's program of “strong objectivity” proposes the idea that starting from the position of the marginalized facilitates our scrutiny of bias that is often buried under supposed impartiality. Precisely because “the standpoint of other, non-European cultures…their scientific and technological needs and desires are not always those of elites in the North,” (21) the investigation of distinct “scientific desires” has the merit to accentuate the difference in value assumptions behind scientific projects. Accordingly, this paper analyzes the invention of New Zealand scientists in response to Iranian buyers and highlights the insights of Malaysian Muslim scientists in relations to Australian and European meat producers. In the long run, this work contributes to efforts that aim to flesh out a global history of veterinary science that recognizes the field's trans-cultural characteristics, and continues to challenge the rigid binaries between the West and the Rest, and between science and culture.

Given the fact that animal suffering is a highly sensitive issue and often stirs up emotional debates or even accusations, pseudonyms are used for the Malaysian scientists at University X, although it is impossible to completely ensure their anonymity. After some long discussion with my interlocutors, we believe that pseudonyms are better in terms of partially protecting their privacy. All the interviews were recorded with the interviewee's agreement, and later transcribed and analyzed.

Two disclaimers should be briefly made here. One is related to the research data regarding MIRINZ I have used in a previous article (22). First, while in my previous publication I theorized the animal body in halal slaughter as a meeting point of various ontologies (23), in this article I stay within the ontology of animal suffering constructed and inhabited by veterinary science. Observing how it deals with the requirement of halal status, I aim at reinvigorating the anthropological interest in animal ethics (24) and facilitating exchanges of ideas between social sciences and veterinary sciences. The other disclaimer is concerned with the issue of bleeding efficiencies under different slaughter methods. The issue itself has generated many interesting scientific experiments and deserves another paper. Without sufficient space to analyze the entire discussion about the blood issue, a quick remark on blood may invoke the stigma of hematophobia and unintendedly obscure the ethical concern in halal slaughter. Given the complexity that the issue of stunning already involves, I decide not to deal with the blood issue here.

Several technical terms regarding stunning are instructive to the discussion that follows. Some elaboration is helpful. As this article will reveal, for sheep and cattle there are two major possible ways to “make stunning halal.” One is head-only electrical stunning, widely used in New Zealand for producing halal lamb meat; the other is non-penetrative mechanical stunning, commonly used in Australia for producing halal beef. The following discussion is arranged into three parts of developments that have had decisive impacts on the evolution of “halal stunning.” They are organized more or less chronologically. Phase one explores the history of obligatory stunning in New Zealand's meat industry. Phase two reconstructs a techno-moral history behind the invention of halal stunning in New Zealand, in which the need to combine obligatory stunning and halal slaughter in order to save the country's economy was given as a task to veterinary scientists. Both Phase one and Phase two are concerned with electrical stunning. Phase Three turns to mechanical stunning in Australia and CO_2_ stunning in Europe, as more recent studies regarding their halal status have emerged. The research of Malaysian veterinary scientists at University X is of particular importance here. I will show how, with their simultaneous concerns with Islamic animal ethics and mainstream animal welfare, these veterinary scientists have potentially challenged long-held assumptions about animals' suffering in the global meat industry.

## Phase 1. The Introduction of Obligatory Stunning: Insensibility as Animal Welfare

Two major challenges faced the New Zealand meat producers in the late 1970s. One was the economic transition. As the British government declared in 1971 that it would reduce its business with New Zealand while increasing its trade with other European countries, the New Zealand government decided to explore new markets (25). Soon after the second oil crisis, it started to make “lamb for oil” deals with Middle Eastern countries. The breakthrough came in October of 1979, when the new Iranian government signed a contract to buy 200,000 tons of lamb over four years (26). The only problem: the meat must be halal (27).

The second challenge was the new legal requirement of pre-slaughter stunning. Following European countries concerned with humane slaughter in the late 1970s, the New Zealand government passed a new law that required all farm animals to undergo pre-slaughter stunning. These two challenges together have led the state and meat industry to turn to veterinary scientists. Now it was inevitable to learn the acceptable way to produce halal meat, while applying the techniques of stunning.

At the time, however, most veterinary scientists had no clue about how to scientifically define stunning animals as “humane,” let alone “halal.” From a technical perspective, electrical stunning had already been used for pig slaughter for several years, but to apply it to sheep was uncommon in the mid-1970s. In fact, pre-slaughter stunning was so rare that the veterinary scientist Blackmore stated that there was probably only one company in all of New Zealand that actually used routine stunning on sheep at that time (28). Nobody knew for sure how to stun animals so that the process could be named “humane,” safe to human workers, and profitable enough that it did not deter meat producers.

The lack of technological knowledge about the practice of stunning was a good illustration of the problem that underlie the two major legal pioneers of humane slaughter globally, the 1933 Slaughter of Animal Act in the UK and the 1958 Humane Slaughter Act in the US. For a long time, there had been advocacy for pre-slaughter stunning by animal rights activists, but the technical details were almost non-existent. In hindsight, that was precisely how the laws were written. The two acts, set apart by time and space, share two common features. First, the core definition of humane slaughter depends upon animals that are rendered insensible. To be humane is to render animals insensible before their death. No measurement is stipulated. Second, religious slaughter is allowed as long as animals are also rendered insensible. In short, even when stunning became mandatory in New Zealand, the technical details and the content of animals' physiological responses were yet to be delineated (29).

As I shall demonstrate below, the technical problem was gradually overcome in the 1980s and 1990s. After the grand-mal seizure was established as the sign that indicated insensibility or unconsciousness of animals, veterinary scientists had to determine the parameters of current, amperage, and Hz suitable for different kinds of animals of different ages (30, 31). In this manner, veterinary scientists could gradually set up the “optimal” parameters so that the slaughter could meet the new law of obligatory stunning in New Zealand.

The halal challenge, however, was a tough one. As mentioned above, the conventional halal slaughter does not allow any form of damage to the animal before the act of halal slaughter, which is defined as a ritual killing of a healthy animal. For Muslim jurists, the beginning of the slaughter process has been defined by the throat cut made while invoking God's blessing and approval. Thus, stunning has been seen as pre-slaughter handling, which is outside of the slaughter proper and cannot replace the cut as the means that takes the life of an animal. Following this logic, the question for Muslim jurists was whether the stunning was a kind of harm or the cause of death. If the answer was yes, stunning would violate the Islamic animal ethics of “no injury” or the taboo of consuming carcasses. Indeed, in the early 1980s it was well-known that head-to-back stunning would cause heart arrest and kill an animal even without further cutting (32). In that case, head-to-back stunning could never be part of the halal procedure of slaughter.

Readers may wonder, isn't meat essentially some sort of carcass? Why does it matter if the animal is killed by the stunning or the cut? For Muslim jurists, however, meat can be defined as “meat” precisely because it is not just any carcass, but a result of a divinely accepted way of taking a life, which requires the cut with God's approval. In contrast, carcasses mean dead animal bodies that are not the result of a legitimate act of slaughter, essentially different from animal bodies that can provide halal meat.

To address the dilemma, scientists from the MIRINZ were asked by the government and meat industry to invent a slaughter procedure that would incorporate pre-slaughter electrical stunning while being considered halal by a recognized certifying authority. The specific mission here was to find a method of stunning that causes insensibility but does not kill and does no harm before the cutting. This task has led to much debate about the definition of harm. While supporters of pre-slaughter stunning treated stunning as a form of protection against the harm of killing, for Muslim jurists the stunning itself threatened to be a kind of harm that caused fatal injuries. To satisfy both sides, what technology can prove that no harm has been done, and what kind of religious authority can approve that technology? The short answer is the method called “reversible stunning.” The longer answer is provided below.

## Phase 2. The Invention of Reversible Stunning: Insensibility and Recoverability as Animal Welfare

As soon as New Zealand started to depend on the Middle East lamb meat market at the end of 1979, MIRINZ veterinary scientists began to systematically develop and distinguish two kinds of electrical stunning. The head-to-back stunning was used for commercial purposes, but it was not used for producing halal meat. This was because this kind of stunning would cause cardiac arrest in animals (30, 31), which would compromise Islamic animal ethics, as we just mentioned. In other words, although the head-to-back method was more convenient for humans to slaughter animals by immobilizing them, it was not used in the production of halal meat. Instead, head-only stunning alone was considered possible for producing halal meat (33), because it would merely cause animals temporary loss of consciousness and no cardiac arrest, and the animal could regain consciousness if the cut was not performed. For a while in the early 1980s, this veterinary view was accepted by the Iranian buyers and Muslim scholars that MIRINZ consulted with.

Yet this “temporary loss of consciousness” worried other veterinary scientists in the early 1980s. Newhook and Blackmore at Massey University were especially concerned that the head-only stunning was not humane enough. They started to systematically use the term “reversible” to describe the state of “insensibility” after animals were stunned (34). Here, “reversible insensibility” was not intended to be a positive description of “halal stunning,” as it later became, but was instead deployed as a concept to protest head-only stunning. Indeed, Blackmore and Newhook conducted a three-part experiment in 1982 and insisted that the insensibility could be reversed precisely because the head-only stunning did not stun the animal enough. Here are some crucial results of the experiments. Newhood and Blackmore discovered that sheep could lose their consciousness in 7 s after the application of head-only electrical stunning, but for cattle, they remained conscious for as long as 60 s (34, 35). Furthermore, cattle might regain consciousness even after the cut was performed, which then constituted inhumane torture (35, 36). Therefore, they concluded, head-only stunning should be avoided as much as possible in favor of head-to-back stunning.

After this three-part experiment was published in the leading journal in the field, MIRINZ was forced to face a dilemma: on one hand, Islamic religious scholars would never approve head-to-back stunning because it killed animals without further cutting; on the other hand, scientists like Newhook and Blackmore considered head-only stunning to be inhumane. Could there be a third way out?

MIRINZ scientists aimed at solving the problem by inventing a kind of head-only stunning that allowed the animal to remain insensible all the way through the slaughtering process but that would also enable the animal to regain consciousness if the cut was not performed. Different species of different ages needed different intensities and durations of electrical stunning. The balance must be delicate. If the electricity was too strong and killed the animal, it would compromise the halal requirement of “no harm”; but if it was too weak to induce the grand-mal seizure (as the standard sign of unconsciousness), it would fail to meet the criteria of humane slaughter.

### The Problem With Insensibility: From the EEG to Neurotransmission

Identifying the onset of insensibility was a difficult task. In Newhook and Blackmore's three-part experiment (34–36), the criteria they used to judge the onset of insensibility of animals was hinged upon electroencephalogram (EEG) amplitudes above 35 μV and below 10 μV. However, MIRINZ scientists found something unusual in Blackmore and Newhook's experiments. They pointed out that according to EEG readings, the bilateral severance of the carotid arteries of unstunned sheep resulted in insensibility in as little as 2–7 s and the trace became isoelectric (unconscious) in 10–43 s, yet the EEG of sheep that were stunned head-to-back did not become isoelectric until after 40–51 s. Why would fully stunned animals lose consciousness later than unstunned ones?

MIRINZ scientists conducted new experiments to get some clues about the application of EEG for the assessment of insensibility. They recorded experiments on sheep under four conditions: sheep that were slaughtered by throat cutting without any stunning, electrically stunned head-only and allowed to recover, electrically stunned head-only followed by throat cutting, or electrically stunned head-to-back without the cutting. After the throat cutting without stunning, the sheep lost consciousness after between 8 and 22 s. The head-only stunned sheep followed by throat cutting reached unconsciousness after 50 s. Surprisingly, in the case of the sheep with head-to-back stunning it took 52 s (37). This was quite counter-intuitive, because the whole-body stunning was assumed to more effectively and quickly lead to the loss of consciousness. To explain this incongruity, Devine et al. suggest that (37).

It is unlikely that head-to-back stunning would prolong sensibility over that of a throat-cut animal; therefore, the unexpectedly prolonged period of apparent sensibility… must be due to other factors.

One of the “other factors,” according to MIRINZ, was that an electrical stun itself already compromised the accuracy of the EEG to measure sensibility, because it caused other physiological changes such as inhibition of breathing (37, 38). This raised some doubts about the use of EEG characteristics established for unstunned animals to interpret the state of sensibility of electrically stunned animals. Another kind of index of insensibility was needed.

MIRINZ scientists then adopted the new method of microdialysis to index the unconscious state of animals (39, 40). Interestingly, despite their previous disagreement, MIRINZ scientists and Blackmore from Massey University jointly created a new experiment. Three days prior to the experiment, they stunned all the animals and waited to see if these animals recovered with reasonable health. On the experiment day, they applied an electrical head-only stun of 400 V and 1.5 ampere to 9 cattle and 6 sheep, and then slaughtered them within 10 s. The result showed that stunning and bleeding hastened the speed of brain death, and no animal retained any consciousness. This time, the proof of unconsciousness was no longer solely dependent on EEG, but also on microdialysis probes to measure the density of glutamate, aspartate, and gamma aminobutyric acid in the somatosensory cortex (41). With the help of microdialysis, MIRINZ could tentatively confirm that with the head-only stunning, animals did lose their consciousness permanently until their deaths, if followed by the proper throat cut.

### Redefining Harm: Charting the Range of Recoverable Seizure

Following the standard grand-mal seizure as the sign of unconsciousness, MIRINZ scientists repeatedly experimented to determine the right intensity and duration, so that the animals could enter the state of seizure but later resume breathing. For example, MIRINZ found that sheep would *not* have seizures if stunned with 1.0 ampere for <0.2 s or longer than 20 s (42). After a series of related experiments, the MIRINZ team was able to set up the first systematically-drawn boundary of “reversible stunning” in 1993. With this range of “reversible stunning,” the leading scientist Gilbert of MIRINZ praised the stunning system as being accepted as “humane to the animal, safe for the workers, virtuous and halal by Muslims worldwide” (43).

Gilbert's confidence did not come from scientific experiments alone. It also came from the Islamic legal opinion produced by an important series of meetings held by Muslim World League and the World Health Organization in the 1980s. The motivation of the Eastern Mediterranean Region of the WHO to hold one particular convention was to address the problem of what they identified as some populations' “voluntary reduction of nutrition by avoiding eating certain types of food, especially meat” (33). One of the reasons for this phenomenon was identified as “religious belief.” In the 1980s, the branch office of WHO was concerned with “nutritional imbalances” of Muslims living or traveling in non-Muslim-majority places or Muslim countries that depended heavily on meat imported from places where halal slaughter was not available or not guaranteed. Hence they launched a series of events to promote “the right path to health” and “health education through religion” (33), which was supported by the WHO, the Food and Agriculture Organization of the United Nations, the Veterinary Institute of the Department of Health in Berlin, and the Muslim World League.

One major mission of a meeting in 1986 was to verify that the proper application of head-only stunning would not cause permanent harm or death to the animal. Scientists and Islamic scholars attended the June 30th to July 3rd convention at the Berlin Institute of Veterinary Medicine, where experiments were conducted on a 35 kg adult sheep and an 18 kg lamb. The two animals were stunned by electricity of 300 V and 1.25 A for 3 s. The animals displayed typical stages of seizures, and then recovered (33). Along with this, the convention also played a video sent from the MIRINZ team, in which a cow of <450 kg was stunned and recovered. The meeting also reviewed other experiments conducted at the University of Edinburgh. Finally, the Muslim World League and the Eastern Mediterranean branch of the WHO issued the “Islamic Ruling on Animal Slaughter,” stating that:

Extensive experience in Western countries and in New Zealand has shown that electric stunning applied to the head only does not cause death and is reversible. The animal so stunned will make a complete recovery if it is not slaughtered.

In the Islamic ruling, the “reversible” state of animals' insensitivity became the evidence of the *harmlessness* of the “reversible stunning” and its compatibility with Islamic animal ethics. Meanwhile, in the scientific experiments that the Muslim jurists reviewed, the state of seizure ensured that the process was humane. This result from the 1986 convention was exhilarating to the New Zealand veterinary scientists. After all, when they started the task, the kinds of stunning they had tried could well be considered neither halal nor humane. Now the head-only electrical stunning was both. Finally, the two problems of insensibility (humanness) and recoverability (halalness) were both solved.

## Phase 3. Rethinking Stunning: Hormones and Hemorrhage

As the international trade with halal commodities continued to grow, the systematization and scientification of halal certification ushered in a new era in the 1990s. The trends of halal standardization became all the more influential in the 2000s and 2010s. A key player of this emerging standardization is the Malaysian government apparatus, which is in charge of all Islamic affairs including halal matters. It has substantial influence in the halal industry and halal market worldwide (44, 45), and its halal standards are followed by hundreds of halal certifying bodies around the world. Importantly, it has recognized head-only stunning as a legitimate way to produce halal meat. In 2006, however, it discovered a potential problem in Australia.

When Malaysian representatives visited Australian slaughterhouses, they found an unreported procedure called thoracic sticking, which was an extra stabbing of the animal after the slaughter cut. This practice worried the Malaysian Islamic jurists, so they immediately consulted veterinary scientists in Malaysia but initially were unable to obtain a clear explanation of the practice. They then turned to consult with veterinary scientists at University X. The scientists explained that thoracic sticking accelerated the dying process and recommended that it was necessary to ensure animal welfare. The Muslim scholars adopted their advice. A potential crisis was averted. Since then, Islamic scholars from the Malaysian government agency and veterinary scientists have maintained their collaboration in responding to newly discovered situations or new requests from international meat traders.

The halal certification for meat production must be renewed every 2 years, with periodic auditing in between the re-certifications. Some Australian livestock traders once wished to change the way they produced halal meat, and they sponsored the research of scientists at University X to learn more about the pain and stress that farm animals suffer during the slaughter. In Dr. Gibran's description, the motivation of the Australian livestock companies was as follows,

[the Australian meat industry], they don't like this non-penetrative [mechanical] stunning. Why? Number one, because there may be mis-stunning…then you repeat the stunning ….. then the carcass becomes *haram* [impermissible, as opposed to halal]. They don't like this, they say they are losing [money], because when it becomes haram the price is lower. So it's not profitable…

…So they prefer to do the [mechanical] penetrative stunning……So that's why they wanted us to do a comparison, [between] the penetrative, [and] non-penetrative to show [if it is possible] that the outcome is more or less similar. [Which according to our research is impossible to achieve]. But the religious council can never accept penetrative stunning, because when you look into penetrative stunning, definitely the animal will suffer brain death, the animal will not recover (Fieldnotes, April 5, 2019).

Although disappointed, the Australian company was willing to accept the reality. A valuable lesson was revealed about the cultural differences in evaluating animal suffering at different stages: it was not enough to simply render animals insensible, because making sure that the animal body was protected from harm before the act of killing was crucial for Islamic animal ethics.

For scientists at University X, however, something more disturbing emerged after this experiment, as they closely examined both the penetrative and non-penetrative stunning in relation to animals' hemorrhage and stress. In what follows, I first discuss the significance of experiments on animal stress, and then move to the shocking revelation of the problem of hemorrhage in the consequence of mechanical stunning.

### Measuring Physiological Stress

Although scientific concerns about farm animals' stress have a long tradition, during the 1970s, “the behavioral measures were preferred to physiological measures and the only debate about stress was semantic” (46). The link between hormones and physiological measures was more pronounced in the later decades (47–49), yet concerns over the difficulties in assessing stress and animal welfare continued (50–52). For example, Broom has called for more precise measurements of how poor animal welfare is in all aspects of housing, transport, slaughter, and so on (52).

During the twenty-first century, studies of stress, hormones and slaughter among farm animals began to figure more prominently (47, 53, 54). This approach helped reshape the old scientific tradition of judging the moral worth of different slaughter methods mainly based on evidence from neurological transmission that indicates insensibility to pain. After all, the notion of pain has intrinsic ambiguities. Scientifically speaking, pain is highly subjective and has no universal index (55), let alone across species and under drastically different circumstances. All the indexes of animal suffering inevitably require interpretation. Additionally, there is the risk that too much focus on pain at the moment of death may inadvertently neglect other problems in the entire life of a farm animal, such as tail-docking, castration and ear-tagging, not to mention inadequate space, restricted access to healthy movement, and concentrated diets. Indeed, even the pre-slaughter handling can pose a tough question: when animals face different slaughter methods, which aspect should be considered worthy of protection? If we can deal with pain, can we also mitigate stress?

As the concern with stress emerged, unprecedented discovery also showed up. In the abovementioned study of cattle's stress in Australia, the Malaysian scientists found that the so-called humane mechanical penetrative stunning caused cattle great physiological stress as reflected in the percentage change of ACTH (adrenocorticotropic hormone) and other hormones (56). In other words, despite being insensible to the pain of the cut, animals do experience extreme stress right before death. The Malaysian scientists wished to compare the level of hormones of cows slaughtered with conventional stunning and those slaughtered using halal methods without stunning, but there was one problem. In Australia, no animal could be slaughtered without stunning. So the Malaysian veterinary scientists could not just conduct halal slaughter alone in their experiment. Hence, a post-cut stunning after halal slaughter was then adopted as a compromise that could at least partially represent the effects of halal slaughter. The result showed that the stress of animals slaughtered with mechanical stunning was higher than the group slaughtered with the modified halal procedure. Nevertheless, Malaysian veterinary scientists repeatedly emphasized to me that the results were not unequivocal, and it might be that the range of analyses available to them was not sufficiently specific to allow a definitive conclusion to be drawn.

Other than giving advice to Australian meat producers, Malaysian veterinary scientists at University X also serve as advisors for some European organizations in matters regarding food production and animal welfare. Given the growing Muslim populations in Europe, commonly raised questions are the halal status of animals receiving gas stunning and water-bath stunning. Due to the limits of this paper, I will briefly dwell on the case of gas-stunning. While applying carbon dioxide (CO_2_) for stunning pigs and poultry is common in Europe, the effect of CO_2_ on different animal species has been a controversial issue.

Supporters of gas stunning highlight the advantages of CO_2_ stunning such as its reduction of human handling prior to slaughter and its ability to cause irreversible unconsciousness. Opponents, on the other hand, insist that a high concentration of CO_2_ causes great pain, fear and stress in animals. As to the possibility of applying gas stunning to rabbits, related scientific knowledge available back in the 2000s was insufficient, and the effects of stunning compared to religious slaughter without stunning was even more limited. One notable work that did compare halal slaughter with electrical stunning was conducted by Lopez et al. (57). In their experiment, they observed that after the halal slaughter, 20 rabbits had no vocalization, spasms or movements, and their bodies remained relaxed and floppy; the 30 rabbits that underwent electrical stunning and standard slaughter procedures (electrical stunning and exsanguination after the cutting) also had no vocalizations or movements before slaughtering, but one rabbit arched and flexed its back for a moment after slaughtering. This work was important to veterinary scientists at University X, who later studied the effects of gas stunning compared to halal slaughter without any stunning on the welfare of rabbits.

In 2014, the Malaysian scientists conducted a new project on rabbits to study their stress. They gathered 80 male New Zealand white rabbits and divided them into two groups of 40 animals. Each group received either gas stunning or halal slaughter without any stunning. By comparing the change of adrenaline, noradrenaline and other blood parameters in the bodies of rabbits under the two different slaughter methods, they wished to more accurately assess the stress that rabbits endure under different conditions.

The result showed that both slaughter methods caused hyperglycemia, or a condition in which an excessive amount of glucose circulates in the blood plasma. More drastically, however, the scientists at University X observed a 5-fold rise in adrenaline among rabbits with halal slaughter and a 10-fold rise among those with gas stunning (58). The noradrenaline was seven times higher in the group that underwent halal slaughter without stunning and twelve times higher for the group that underwent gas stunning (58). Also, the group of rabbits stunned with gas (GS) exhibited significantly higher levels of blood glucose than the group that underwent halal slaughter, which indicated a greater level of stress because there is higher energy metabolism during stressful situations (59). In short, based on the indexes of hormones and of hyperglycemia, gas stunning can be more stressful than halal slaughter without stunning according to this experiment. Despite this discovery, all the Malaysian scientists have been very cautious about this finding. As Dr. Gibran expressed to me,

When we collect the blood at the point of slaughter, the level of hormones can be very high. We have to be cautious because high levels of hormones can be due to pre-slaughter stress, not really because of the neck cut. It can be cumulative, can be due to the slaughtering process itself, can be due to the stunning… so it's actually very difficult to categorize them. Because the stress can be cumulative, for example you had a cut in your hand, then you had to walk under the hot sun, you have double stress (fieldnotes, April 5, 2019).

The Malaysian veterinary team tried their best to rule out all these possible unwanted influences, and discovered the indication of great stress caused by conventional humane slaughter. It seems that after the cut, both methods lead to an increase in critical blood constituents like catecholamines, lactate, glucose, calcium, magnesium, and proteins. These biochemical and hematological changes in rabbits indicated an intense stress response from the animals in order to cope with this situation.

While Malaysian scientists all support pre-slaughter stunning, they are also concerned with methods of reducing stress. They have been trying to study further about animal stress beyond a narrow concern with the pain of the cut. Dr. Gibran expressed to me his sympathy with animals who received gas-stunning in Europe: “Among mechanical, electrical…I don't like gas-stunning. It is very painful.”

### Mechanical Stunning Causing Hemorrhage

Other than pain and stress, the principle in Islamic animal ethics of “no harm” before the act of slaughter continues to be a primary driver of new scientific research for veterinary scientists at University X. Indeed, ever since they started to serve as the scientific advisors to the religious council inside the government agency, the Malaysian scientists realized that there was a lack of locally generated experimental data that actually included the recovery test after the non-penetrative stunning. In the words of Dr. Hafiz, in the past when the government agency that administers religious affairs made decisions about halal stunning,

The fatwa^1^ was based on the information provided by Western countries. So technically, the fatwa council accepted the non-penetrative mechanical percussive stunning [as halal], why? Because technically, it's blunt, unlike penetrative—you know sharper—blunt so it shouldn't crack or shouldn't penetrate. Through permissible degrees of power (Fieldnotes, April 4, 2019).

In other words, over the past few decades the halal status of mechanical non-penetrative stunning was based on assumptions rather than solid scientific evidence. The assumption that animals could recover from mechanical non-penetrative stunning had never been scientifically established, unlike the comprehensive research in the case of reversible head-only electrical stunning. Dr. Hafiz explained to me,

Every two or three years the religious council will go to the Australia and New Zealand abattoir, because we import beef and lamb from Australia and New Zealand and they are doing [electrical or mechanical] stunning. That's why it becomes very important. When you do non-penetrative stunning we check the skull, ok, the skull looks intact. No crack. When the skull is intact we assume that. But [in Australia and New Zealand] we will never be allowed to wait and see [if the stunned animal can actually recover their consciousness]… They say it's prohibited, once the animal is stunned, the animal should be slaughtered right away (Fieldnotes, April 4, 2019).

Given the legal restrictions in Australia and New Zealand, it is no wonder that Malaysian scientists must conduct research in Malaysia in order to find more evidence. While they have no problem with rendering the animal insensible to the pain of being killed, they care deeply about the Islamic ethics of no harm and hence the recoverability of farm animals. As Dr. Gibran told me,

We only accept the non-penetrative [stunning] because we believe that animals can recover from non-penetrative stunning, but our recent study shows otherwise (Fieldnotes, April 5, 2019).

In all the related experiments they conducted in recent years, they asked questions like: After it is rendered insensible to pain with mechanical non-penetrative stunning, can the animal still regain consciousness and resume life as normal? Is the animal actually stunned to death, even though the Australian producers claim otherwise? Dr. Hafiz said the following,

……in our study, even the lowest psi, we have brought down the lowest psi to 120, still we encounter skull fractures. We were very surprised even though the skull appears very clear. When we brought the skull to the lab, we found that the brain was severely hemorrhaged.……I don't think that the animal will recover. Because whether [the stunning is] penetrative or non-penetrative, the animal will suffer from brain death. So I don't think the animal will recover (Fieldnotes, April 4, 2019).

On the basis of their recent research, Malaysian scientists conclude that animals do suffer hemorrhage or even brain death after receiving mechanical non-penetrative stunning.

Dr. Hafiz recalled this memory and showed discomfort while I sat with him in one of their conference rooms at University X, before we departed for their laboratory farm. After some discussion, we felt that the source of discomfort was multiple and complicated. Put it simply, these concerns were scientific and Islamic at the same time.

On the one hand, after the laboratory work that the research team at University X had done, they now knew that even blunt, non-penetrative stunning with low power could cause hemorrhage or even brain death. So for them, this means that for all these years, the so-called mechanical halal stunning has actually not been halal because it hurts animals before the act of slaughter. Even though any Muslim who unknowingly consumes non-halal food is immediately forgiven by God and should not feel guilty, the reality of learning the truth through scientific studies was hard to swallow.

On the other hand, while animal farms in New Zealand and Australia always claim that they were providing halal meat and conducting halal stunning, legally they could not prove it by showing the auditors that the stunned animals could actually recover and resume normal social life. This can lead to some ethical confusion, because the abattoir was supposed to scientifically prove what they claimed to be religiously approved. Here, the feeling of discomfort is a complicated product of scientific know ability, multiple moral responsibilities, and legal restrictions.

## Discussion: Rethinking Animal Suffering with Multiple Animal Ethics

This paper reconstructs a brief socio-technological history of halal slaughter through scientific research on animal suffering. Veterinary science, animal welfare, and Islamic animal ethics are equally indispensable in this process. It was due to the “no harm” principle in Islamic animal ethics that New Zealand scientists charted the possible range of insensibility and recoverability. For the same reason, Malaysian scientists expanded their concern from identifying insensibility and recoverability to comparing levels of stress, bone fracture and hemorrhage that animals endure.

The existence of multiple considerations about animal suffering has been an important motivation for scientists to experiment on new terrains, but the multiplicity itself often does not automatically translate into a rapid change in the established system. This point is evident in the mainstream system of meat production and consumption. For example, scholars have found that global consumers of meat nowadays often focus on the “naturalness” of the animals' living conditions (60–63), whereas livestock producers tend to focus on meeting basic health requirements (60, 61, 64). In fact, farmers even tend to positively link fast and efficient growth to good animal welfare. The trend in the farming industry is still geared toward more indoor sheltering and concentrated diets, despite the emergence of some smaller farmers and alternative farming methods. We are still far away from challenging the dominance of concentrated, productionist-oriented animal farms.

Neither supporters of secular animal welfare nor Islamic animal ethics wish to see the mistreatment of farm animals. While a large number of European consumers have boycotted halal meat because they believe that it is a product of cruelty (65), many of them do not reject secular meat products produced from farm animals that have life experiences that raise welfare concerns (66). There is still a wide space of improvement when it comes to overly crowded pig farms, the high percentage of cattle that are lame and diseased, as well as lamb's suffering from cold, footrot, and husbandry procedures (67, 68). Numerous campaigns have been mounted to ban religious slaughter, yet few beyond the most committed activists think it plausible to call for a ban on capitalist animal factories, which seems unlikely to become a populist movement.

Against the phenomena above, a few interrelated points can be summarized again about ways of rethinking animal suffering with multiple ethics. First, a more empathetic understanding of different modes of compassion toward animals helps us to reflect more deeply on the operations of abattoirs. Second, the “no harm” principle that Islamic animal ethics cherishes is in principle compatible with contemporary animal welfare concerns, despite contrary stereotypes. Third, the moment of death is often disproportionately targeted as the determiner of the cruelty or humaneness of meat production methods. Last, studying trans-cultural collaboration for veterinary practices can expose blind spots in conventional thinking and contribute to exploring animals' suffering from broader perspectives.

## Conclusion

As the history of veterinary science continues to grow into a rich, multi-disciplinary field (8, 69–72), the emerging studies in veterinary anthropology can bring further insights into the relations between sciences, cultures, and animals. Here, it is paramount to remind the readers that neither “culture” nor “religion” is a fixed thing in the real world. Instead they are flows of meaning and embodied knowledge produced out of shifting techno-moral landscapes (73–75). In the cases that this paper delineates, it was precisely the flexibility of Islamic practices—under the guidance of Islamic compassion toward animals—that has helped generate innovative scientific research on animal's suffering. These complex developments encourage us to keep investigating the different ways in which veterinary science is always already tied to multiple value systems. It allows anthropology to be culturally reflective regarding the practices of veterinary science and to imagine how these practices may be otherwise.

## Data Availability Statement

The original contributions presented in the study are included in the article/Supplementary Material, further inquiries can be directed to the corresponding author.

## Ethics Statement

The studies involving human participants were reviewed and approved by Yen-Ping Lin, National Cheng Kung University Governance Framework for Human Research Ethics. Case Number: 107-196. Written informed consent for participation was not required for this study in accordance with the national legislation and the institutional requirements.

## Author Contributions

The author confirms being the sole contributor of this work and has approved it for publication.

## Funding

This research was funded by Taiwan's Ministry of Science and Technology (110-2410-H-110-053-MY2 and 107-2410-H-110-070-MY3).

## Conflict of Interest

The author declares that the research was conducted in the absence of any commercial or financial relationships that could be construed as a potential conflict of interest.

## Publisher's Note

All claims expressed in this article are solely those of the authors and do not necessarily represent those of their affiliated organizations, or those of the publisher, the editors and the reviewers. Any product that may be evaluated in this article, or claim that may be made by its manufacturer, is not guaranteed or endorsed by the publisher.

## Footnotes

^1^Religious ruling or legal advice in Islamic communities. In different historical periods and different areas, a fatwa may not have any binding effects. A fatwa can be a mobilizing force, however. In modern times, some fatwa are directly linked to legal regulations, such as halal certification and halal standards set up by a government agency that administers religious affairs.
